# Clinical Impact of Pretreatment Human Immunodeficiency Virus Drug Resistance in People Initiating Nonnucleoside Reverse Transcriptase Inhibitor–Containing Antiretroviral Therapy: A Systematic Review and Meta-analysis

**DOI:** 10.1093/infdis/jiaa683

**Published:** 2020-11-17

**Authors:** Silvia Bertagnolio, Lucas Hermans, Michael R Jordan, Santiago Avila-Rios, Collins Iwuji, Anne Derache, Eric Delaporte, Annemarie Wensing, Theresa Aves, A S M Borhan, Alvin Leenus, Neil Parkin, Meg Doherty, Seth Inzaule, Lawrence Mbuagbaw

**Affiliations:** 1 Global HIV, Hepatitis and STI Programmes, World Health Organization, Geneva, Switzerland; 2 Virology, Department of Medical Microbiology, University Medical Center Utrecht, Utrecht, The Netherlands; 3 Wits Reproductive Health and HIV Institute, University of the Witwatersrand, Johannesburg, South Africa; 4 Department of Public Health and Community Medicine, Tufts University School of Medicine, Boston, Massachusetts, USA; 5 Division of Geographic Medicine and Infectious Disease, Tufts Medical Center, Boston, Massachusetts, USA; 6 Tufts Center for Integrated Management of Antimicrobial Resistance, Boston, Massachusetts, USA; 7 Centro de Investigación en Enfermedades Infecciosas, Instituto Nacional de Enfermedades Respiratorias, Mexico City, Mexico; 8 Department of Global Health and Infection, Brighton and Sussex Medical School, University of Sussex, Falmer, United Kingdom; 9 Africa Health Research Institute, KwaZulu-Natal, South Africa; 10 TransVIHMI, University of Montpellier, Institut de Recherche pour le Développement, Institut national de la santé et de la recherche médicale, Montpellier, France; 11 Department of Health Research Methods, Evidence and Impact, McMaster University, Hamilton, Ontario, Canada; 12 Data First Consulting, Sebastopol, California, USA

**Keywords:** pretreatment HIV drug resistance, HIV drug resistance, virological failure, treatment failure, ART, NNRTIs

## Abstract

**Background:**

Increased access to antiretroviral therapy (ART) has resulted in rising levels of pretreatment human immunodeficiency virus drug resistance (PDR). This is the first systematic review and meta-analysis to assess the impact of PDR on treatment outcomes among people initiating nonnucleoside reverse transcriptase inhibitor (NNRTI)–based ART, including the combination of efavirenz (EFV), tenofovir (TDF), and lamivudine or emtricitabine (XTC).

**Methods:**

We systematically reviewed studies and conference proceedings comparing treatment outcomes in populations initiating NNRTI-based ART with and without PDR. We conducted subgroup analyses by regimen: (1) NNRTIs + 2 nucleoside reverse transcriptase inhibitors (NRTIs), (2) EFV + 2 NRTIs, or (3) EFV/TDF/XTC; by population (children vs adults); and by definition of resistance (PDR vs NNRTI PDR).

**Results:**

Among 6197 studies screened, 32 were analyzed (31 441 patients). We found that individuals with PDR initiating NNRTIs across all the subgroups had increased risk of virological failure compared to those without PDR. Risk of acquisition of new resistance mutations and ART switch was also higher in people with PDR.

**Conclusions:**

This review shows poorer treatment outcomes in the presence of PDR, supporting the World Health Organization’s recommendation to avoid using NNRTIs in countries where levels of PDR are high.

## Teaser

Higher risks of virological failure, acquisition of new resistance mutations, and treatment switch were observed among adults and children with pretreatment HIV drug resistance initiating nonnucleoside reverse transcriptase inhibitor–containing regimens, including efavirenz/tenofovir/lamivudine or emtricitabine.

As of June 2019, >24 million people were receiving antiretroviral therapy (ART). Expanded access to ART has been associated with a rise in human immunodeficiency virus (HIV) drug resistance (HIV-DR), which in turn is predicted to be associated with increased mortality, program cost, and HIV incidence [1].

Pretreatment HIV-DR (PDR) can be transmitted at the time of HIV infection or acquired by virtue of prior antiretroviral (ARV) drug exposure(s). Irrespective of its origin, population-level information about the ARV drugs to which HIV is resistant and its potential impact is critical to guide selection of effective therapy. Nationally representative PDR surveys yield results reflecting the programmatic reality of a public health approach for HIV treatment, whereby the same first-line regimen is offered to all individuals being treated irrespective of prior use of ARVs and without a documented history of virological failure (VF) or presence of resistance.

Robust available data have shown increasing PDR prevalence to nonnucleoside reverse transcriptase inhibitors (NNRTIs) in low- and middle-income countries (LMICs) [2, 3]. In resource-rich settings, HIV-DR testing is recommended at time of HIV diagnosis or prior to treatment initiation, whereas in LMICs this is not a standard practice due to cost, infrastructure, and limited capacity. In these settings, population-level PDR surveillance is essential to guide optimal first-line regimen selection [4, 5].

Due to the high PDR levels in several countries and because of several benefits of dolutegravir (DTG), the World Health Organization (WHO) recommended DTG-containing ART as the preferred first- and second-line regimen [6]. While many LMICs are transitioning from NNRTI- to DTG-containing regimens, some settings or populations have limited access to DTG due to various considerations.

The clinical relevance of PDR among people starting NNRTI-based ART has been assessed in various studies, at times with conflicting results [7, 8]. This is the first systematic review and meta-analysis to assess the impact of PDR on treatment outcomes among people initiating NNRTI-containing ART, with a focus on populations initiating efavirenz (EFV), irrespective of the nucleoside reverse transcriptase inhibitor (NRTI) backbone, and in populations initiating EFV in combination with lamivudine or emtricitabine (XTC) and tenofovir disoproxil fumarate (TDF). Findings from this review directly informed the WHO Guidelines on the Public Heath Response to Pretreatment HIV Drug Resistance [5], and provided supporting evidence to the WHO’s 2019 updated recommendations on first- and second-line ART [6].

## METHODS

We performed a systematic review and meta-analysis of studies published from 1 January 1989 to 31 August 2019 assessing the impact of PDR on ART outcomes. Studies were included if they (1) included people living with HIV initiating or reinitiating NNRTI-based ART; (2) reported genotypic HIV-DR results generated by Sanger or other methods with full coverage at known HIV-DR–associated codons using a 20% sensitivity cutoff; or (3) reported correlation between PDR and at least 1 of the following primary treatment outcomes: VF, virological success (VS), time to VF or VS, death, composite outcome of VF or death; or secondary outcomes: new HIV-DR mutations, ART interruption, or switch to non-NNRTI-based regimens. Supplementary Appendix 1 provides additional information on search strategy and data extraction procedures.

We conducted subgroup analyses by regimen: (1) NNRTIs (nevirapine [NVP] and/or EFV) + 2 NRTIs (32 studies), (2) EFV + 2 NRTIs (subset of 8 studies), or (3) EFV/XTC/TDF (subset of 6 studies); by population (children; adults); and by definition of the exposure (PDR; NNRTI PDR with or without NRTI PDR). Table 1 describes the characteristics of studies included in the subgroup analysis.

**Table 1.** Main Characteristics of Included Studies Assessing Impact of Pretreatment Human Immunodeficiency Virus Drug Resistance Among People Starting Nonnucleoside Reverse Transcriptase Inhibitor–Based Antiretroviral Therapy

						Subgroup Analysis			
Study, First Author (Publication Year)	Country	Population	Reported Prior Exposure to ARV Drugs Before ART Started (% ARV Drug Exposed)	PDR Prevalence in the Study Population, %	Sample Size (No. of Genotypes)	EFV- Based ART	EFV/XTC/TDF	Sample Size of Pts on EFV/XTC/ TDF^a^	Definition of VF^b^
Avila-Rios (2016) [13]^c,d^	Mexico	Adults	None or NR	15.5%	264	✓	✓	160^e^	50 c/mL
Bannister (2008) [14]	Europe, Israel, and Argentina	Adults	None or NR	11.4%	525	…	…	…	…
Bansi (2010) [15]	United Kingdom	Adults	None or NR	9.9%	1175	…	…	…	…
Boender (2015), PASER-M cohort [42]	Kenya, Nigeria, South Africa, Uganda, Zambia, Zimbabwe	Adults	Mixed (5%)	13.8%	2579	…	…	…	…
Boerma (2016) [16]	Nigeria	Children^f^	None or NR	15.9%	82	…	…	…	…
Borroto-Esoda (2007) [17]	Canada, Puerto Rico, United States, Argentina, Brazil, Chile, Mexico, France, Germany, United Kingdom	Adults	None or NR	16.5%	546	✓	…	…	400 c/mL
Chaix (2007) [18]	France	Adults	None or NR	13.1%	350	…	…	…	…
Clutter (2016) [19]	United States	Adults	None or NR	NR	3245	…	…	…	…
Coelho (2018) [20]	Brazil	Adults	None or NR	10.9%	596	…	…	…	…
Crowell (2015) [21]	Mali	Children^g^	Mixed (12%)	25.8%	120	…	…	…	…
Derache (2019) [8]^c,d^	South Africa	Adults^h^	None or NR	8.7%	837	✓	✓	812	<50 c/mL
Inzaule (2019), PASER-M cohort [43]	Kenya, Nigeria, South Africa, Uganda, Zambia, Zimbabwe	Adults	Mixed	4.3%	530	…	✓	…	…
Hamers (2012) [23]^c^, PASER-M cohort	Kenya, Nigeria, South Africa, Uganda, Zambia, Zimbabwe	Adults	Mixed (4%)	6.7%	2579	✓	…	530^i^	1000 c/mL
Hermans (2019) [40]^c^	South Africa	Adults	Mixed (6.3%)	12.9%	194	✓	✓	148	1000 c/mL
Hong (2015) [24]	Namibia	Adults	Mixed (21%)	6.8%	384	…	…	…	…
Kantor (2015)^ [25]j^	Brazil, Haiti, India, Malawi, Peru, South Africa, Thailand, United States, Zimbabwe	Adults	None or NR	7.1%	466	✓	✓	144	2 VL >1000 c/mL
Kityo (2017) [26]	Uganda	Children^k^	None or NR	16.9%	256	…	…	…	…
Kuritzkes (2008) [28]^j^	United States	Adults	None or NR	NR	342	✓	…	…	2 VL ≥200 c/mL
Lai (2012)^ [27]j^	Taiwan	Adults	None or NR	6.9%^l^	1349	…	…	…	…
Lee (2014) [29]	Uganda	Adults	None or NR	3.6%	572	…	…	…	…
Li (2015) [30]	China	Adults	None or NR	<0.5%	444	…	…	…	…
Lockman (2010) [31]	South Africa, Kenya, Zimbabwe, Botswana, Zambia, Malawi, Uganda	Adults	Women preexposed to sdNVP (100%)	NR	241	…	…	…	…
McCluskey (2018) [32]	Uganda	Adults	None or NR	3.5%	738	…	…	…	…
NAMSAL ANRS 12313 (2019) [22]^c,d^	Cameroon	Adults	None or NR	10%	302	✓	✓	302	1000 c/mL
Ngo-Giang-Huong (2016) [33]	Europe, Africa, Asia	Children	Mixed (% NR)	7.8%	476	…	…	…	…
Palumbo (2010) [34]	Botswana, Brazil, India, Kenya, Malawi, South Africa, Thailand, United States, Zimbabwe	Children (aged 6–36 mo)	All but 5 sdNVP exposed	12.2%	148	…	…	…	…
Phanuphak (2014) [35]	Thailand, Indonesia, Malaysia, Hong Kong, Philippines	Adults	None or NR	4.1%	1471	…	…	…	…
Shet (2015) [36]	India	Adults	None or NR	4.3%	599	…	…	…	…
Taniguchi (2012) [37]	United States	Adults	None or NR	16.9%	801	…	…	…	…
Thao (2018) [41]	Vietnam	Adults	None or NR	5.7%	564	…	…	…	…
Wittkop (2011) [38]	Europe, Africa, Asia	Adults and children	None or NR	9.5%	10 056	…	…	…	…
Zu Knyphausen (2014) [39]	Germany	Adults	None or NR	16.1%	1667	…	…	…	…

Abbreviations: ANRS, Agence Nationale de Recherche contre le Sida; ART, antiretroviral therapy; ARV, antiretroviral; c/mL, copies per milliliter; EFV, efavirenz; NAMSAL ANRS, New Antiretroviral and Monitoring Startegies in HIV-Infected Adults in Low-Income Countries; NR, not reported; PASER- M, PharmAccess African Studies to Evaluate Resistance Monitoring; PDR, pretreatment drug resistance; sdNVP, single-dose nevirapine; TDF, tenofovir disoproxil fumarate; VF, virological failure; VL, viral load; XTC, lamivudine or emtricitabine.

^a^Authors of studies including patients receiving multiple nucleoside reverse transcriptase inhibitor backbone were contacted and provided data of patients on EFV/XTC/TDF using standardized definition of resistance, as described in the Methods.

^b^The VL cutoff value used for the EFV/XTC/TDF subanalysis is >1000 copies/mL for all studies (including Avila-Rios et al) except Derache et al.

^c^Authors provided data for this analysis on the impact of nonnucleoside reverse transcriptase inhibitor (NNRTI) PDR among people on EFV-based ART using standardized definitions described in the Methods.

^d^Authors provided data for this analysis on the impact of NNRTI PDR among people on EFV/XTC/TDF using standardized definitions described in the Methods.

^e^Children <18 years of age.

^f^Children <13 years of age.

^g^Children <10 years of age.

^h^Adults >16 years of age.

^i^This subanalysis was published by Inzuale et al, Clin Infect Dis 2019; 68:2158–60.

^j^Case-control studies.

^k^Children <12 years of age.

^l^Children between 6 and 36 months of age.

To increase comparability of the findings, authors of studies assessing impact of PDR in patients receiving NNRTIs were invited to reanalyze their dataset using our study definition of resistance, and focusing to subpopulations receiving EFV + 2 NRTIs and, where available, EFV/XTC/TDF.

### Definitions of Resistance and Virological Failure

In our analysis, PDR was defined by the presence of 1 or more HIV-DR mutations. NNRTI PDR was defined by the presence of 1 or more mutations conferring resistance to NNRTIs, with or without NRTI-associated resistance mutations. Variation in drug resistance interpretation systems used by the study authors was allowed as long as they were congruent with publicly available guidance or resources [9–12].

To increase comparability across studies, the VF threshold was defined as viral load (VL) ≥1000 copies/mL; for studies not reporting results using this threshold, we used the threshold of VF reported by the author.

## DATA ANALYSIS

### Assessment of Risk of Bias

This systematic review is reported according to Preferred Reporting Items for Systematic Reviews and Meta-Analyses (PRISMA). Two investigators assessed the methodological quality of all studies with the Newcastle-Ottawa Scale.

### Statistical Analysis

The primary outcome of the analysis was VF. Studies providing data on the composite outcome of VF or death were pooled with studies providing VF outcomes, as well as analyzed separately. Secondary outcomes were death, discontinuation of ART, switch to a non-NNRTI-based regimen, and incident drug resistance mutations. Studies reporting data using time-to-event outcomes (eg, time to failure) were converted to binary outcomes (eg, VF). Hazard ratios and odds ratios were converted from positive (ie, virological success) to negative (ie, virological failure) for pooling to reflect the relative effect of having PDR versus not having PDR.

We pooled similar measures of effect, where appropriate, in a random effects meta-analysis. We used the generic inverse variance approach to incorporate adjusted effect estimates. Statistical heterogeneity was assessed using the χ ^2^ test for homogeneity with a level of significance α = .10 and the *I*^2^ statistic to quantify inconsistency. Publication bias was assessed using a funnel plot for outcomes with 10 or more studies.

We analyzed the data using Stata/IC 16.0 for Windows and WINPEPI, and present the results as effect measures and 95% confidence intervals (CIs). Additional information on data analysis is shown in Supplementary Appendix 1.

## RESULTS

**Figure 1.**
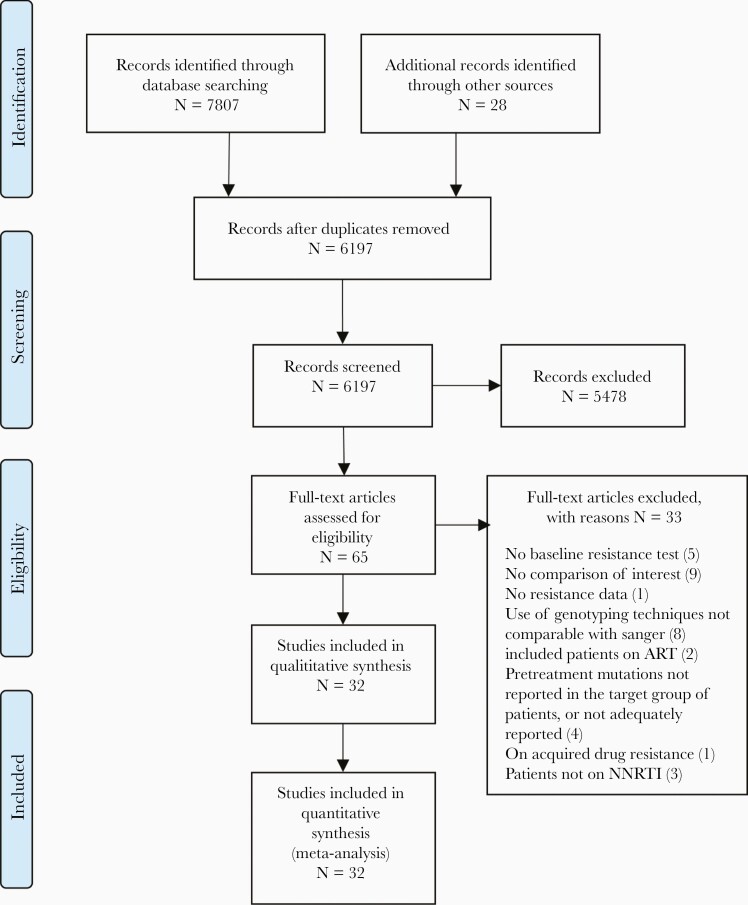
Study selection procedure. Abbreviations: ART, antiretroviral therapy; NNRTI, nonnucleoside reverse transcriptase inhibitor.

The study selection procedure is illustrated in Figure 1. The literature search yielded 7807 studies; an additional 28 articles were identified by experts in the field. We screened 6197 studies after removing duplicates. Sixty-five articles were downloaded for full text screening, of which 32 (30 unique data sets) were included. Twenty-nine were cohort studies, and 3 were case-control studies. Combined, these studies reported data on 31 441 patients. Table 1 summarizes the main characteristics of the included studies. Excluded studies (n = 33) and the reasons for exclusion are reported in Supplementary Appendix 2.

Overall, 30 studies reported on the VF outcome [8, 13–41], 3 studies on treatment switches or discontinuation [13, 28, 42], 2 studies on death [28, 42], and 2 studies on new resistance mutations [23, 26].

**Figure 2.**
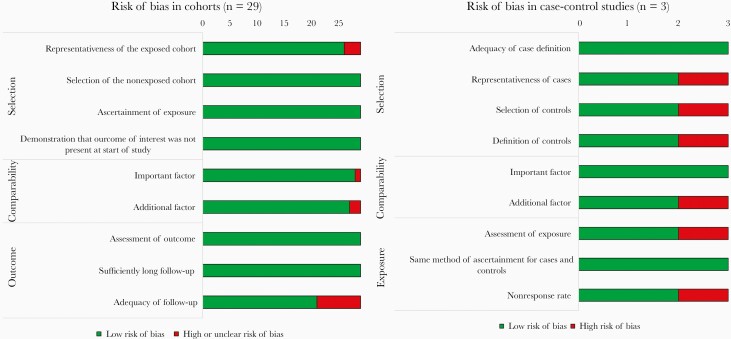
Summary of risk of bias of included studies.

Five studies specifically assessed PDR in children [8, 22, 23, 40, 41]. Eight studies assessed impact of PDR in individuals starting EFV + 2 NRTIs, and 6 studies among people starting EFV/XTC/TDF. Table 1 describes the characteristics of studies included in the subgroup analysis. A summary of the risk of bias is provided in Figure 2 (Supplementary Appendix 3).

For the outcome of VF, we determined that there was publication bias based on funnel plot asymmetry and a significant Egger test (β = 2.69; SE of β = 0.364; *P* < .001).

### Impact of PDR on Treatment Outcomes in People Initiating NNRTI-Containing ART

#### Primary Outcomes

Thirty studies reported the impact of PDR on VF in populations receiving NNRTI-based regimens, containing either NVP, EFV, or a mix of the 2 drugs. Among the 30 studies, 26 defined the outcome as VF (23 studies in adults; 3 in children) [8, 13, 14, 17–20, 22–27, 29, 30, 32–41], while 4 studies reported the composite outcome of VF or death (2 studies in adults, 2 in children) [16, 21, 28, 31].

**Figure 3.**
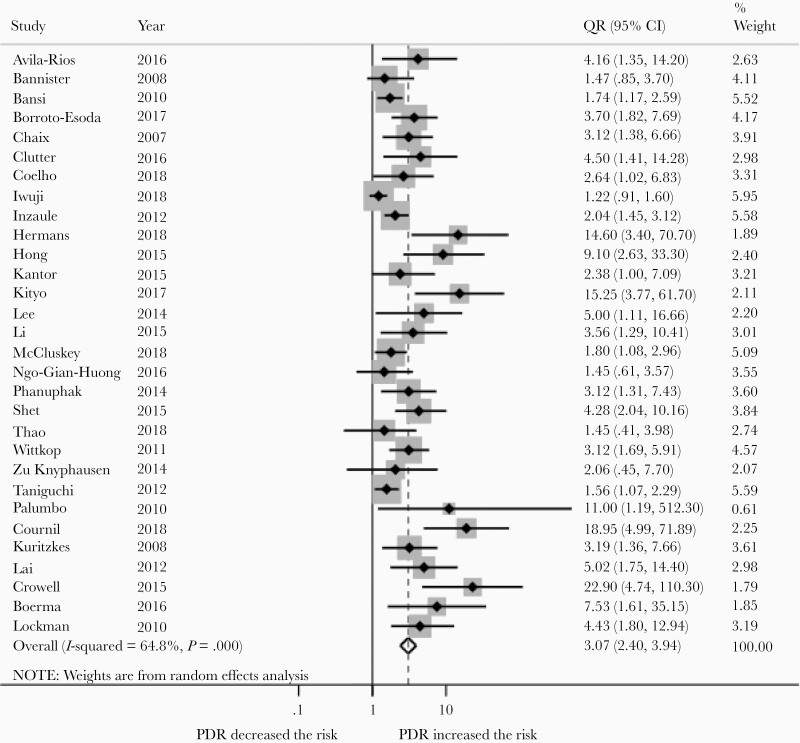
Risk of virological failure (VF) among people with pretreatment HIV drug resistance (PDR) compared to people without PDR initiating NNRTI-based antiretroviral therapy. The dotted vertical line represents the overall risk of VF if PDR is present. The open diamond represents the overall risk of VF if PDR is present. Abbreviations: CI, confidence interval; OR, odds ratio.

Overall, the risk of VF was higher in patients with PDR (odds ratio [OR], 3.07 [95% CI, 2.40–3.94]; Figure 3), and remained so in adults (OR, 2.78 [95% CI, 2.19–3.53]; 25 studies) and children (OR, 7.47 [95% CI, 2.12–26.41]; 5 studies). Heterogeneity was substantial (*I*^2^ = 64.8; *P* < .001). In stratified analysis, the impact remained significant irrespective of the definition of the outcome (ie, VF; VF or death).

**Figure 4.**
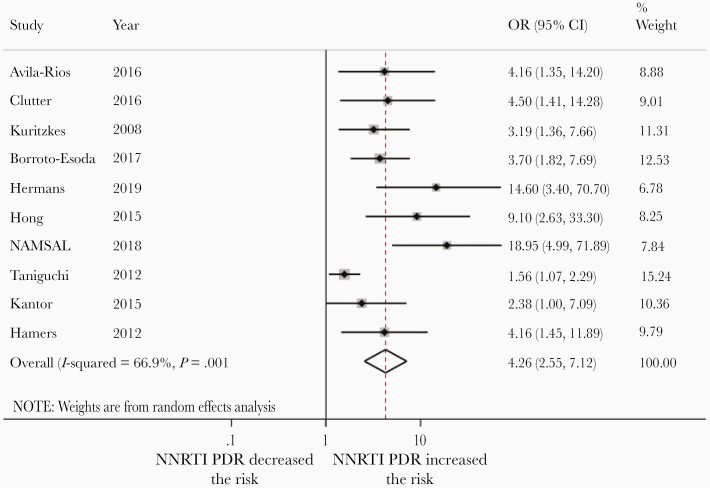
Risk of virological failure (VF) among people with NNRTI pretreatment HIV drug resistance (PDR) compared to people without PDR initiating NNRTI-based regimens. The dotted vertical line represents the overall risk of VF if NNRTI PDR is present. The open diamond shows the confidence intervals of the overall risk of VF if NNRTI PDR is present. Abbreviations: CI, confidence interval; OR, odds ratio.

In sensitivity analysis restricted to 10 studies only focusing on NNRTI PDR among adults, NNRTI PDR was associated with an even more pronounced risk of VF (OR, 4.26 [95% CI, 2.55–7.12]; Figure 4).

#### Secondary Outcomes

**Figure 5.**
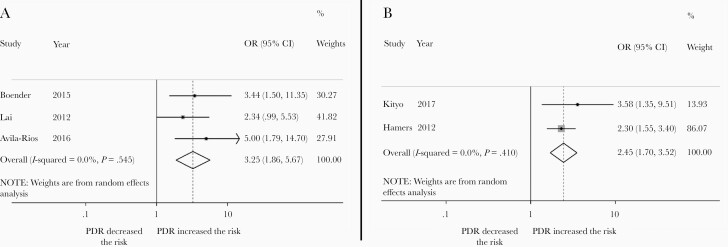
A, Risk of acquisition of new resistance mutations in people with pretreatment HIV drug resistance (PDR) compared to people without PDR initiating NNRTI-based regimens. B, Risk of HIV treatment discontinuation or switch among people with pretreatment HIV drug resistance (PDR) compared to people without PDR initiating NNRTI-based regimens. Abbreviations: CI, confidence interval; OR, odds ratio.

New resistance mutations were more likely to emerge in people taking NNRTI-based first-line ART who had PDR at treatment initiation compared to those without (OR, 2.45 [95% CI, 1.70–3.52]; 2 studies; Figure 5A). ART discontinuation or switch was more likely in people with PDR compared to people without (OR, 3.25 [95% CI, 1.86–5.67]; 3 studies; Figure 5B). There was no difference in the odds of death (OR, 0.89 [95% CI, .31–2.59]; 2 studies) in people with PDR compared to those without.

## SUBGROUP ANALYSIS

We performed subgroup analysis to assess the impact of PDR among populations receiving EFV-based ART [8, 13, 17, 22, 23, 25, 27, 40] and in populations receiving EFV/TDF/XTC (Table 1). Principal investigators were contacted; all but 3 submitted data for this subanalysis using the study definition of resistance described above.

### Impact of PDR on VF in People Initiating EFV + 2 NRTIs

A subset of 8 studies assessed the impact of PDR on VF in adults initiating a regimen containing efavirenz and 2 NRTIs, irrespective of type of NRTI drug used (Table 1) [8, 13, 17, 22, 25, 27, 40, 43]. Meta-analysis revealed an increased risk of VF in patients with PDR or NNRTI PDR (OR, 3.77 [95% CI, 1.96–7.28]; 8 studies; *I*^2^ = 80.2%).

### Impact of PDR on VF in People Initiating EFV/XTC/TDF

**Figure 6.**
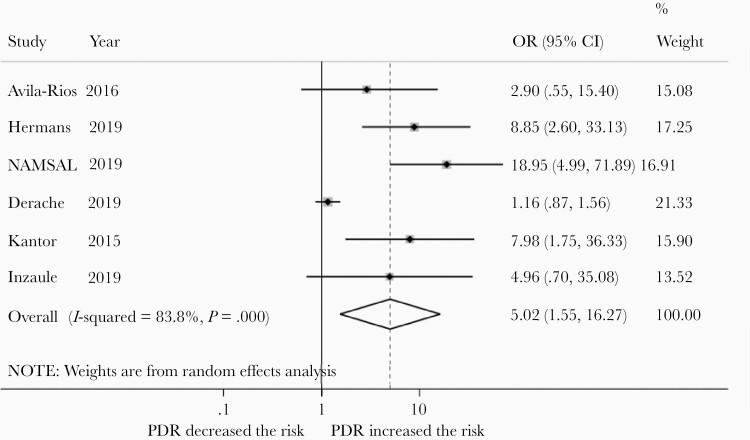
Risk of virological failure in patients with NNRTI pretreatment HIVDR (PDR) compared to those without PDR receiving efavirenz in combination with lamivudine/emtricitabine and tenofovir. Abbreviations: CI, confidence interval; OR, odds ratio.

A subset of 6 studies analyzed outcomes in adults receiving a once-daily, fixed-dose combination of EFV/XTC/TDF (Table 1) [8, 13, 22, 25, 40, 43]. Meta-analysis of these studies revealed that the risk of VF is increased in patients with PDR (OR, 4.48 [95% CI, 1.46–13.68]; 6 studies; *I*^2^ = 82.0%). In a sensitivity analysis focusing on NNRTI PDR, NNRTI PDR was associated with a similar increase in VF (OR, 5.02 [95% CI, 1.55–16.27]; 6 studies; *I*^2^ = 83.8.0%; Figure 6) [8, 13, 22, 25, 40, 43].

## DISCUSSION

Thirty-two studies comprising 30 unique datasets and >30 000 participants were included in this analysis documenting that PDR, and NNRTI PDR in particular, defined as the presence of 1 or more mutations conferring resistance to NNRTIs (irrespective of whether or not NNRTI-associated resistance mutations were paired with NRTI-associated mutations), predisposes to VF, selection of additional drug resistance mutations, and treatment discontinuation or switch. People with PDR were 3 times more likely to experience VF, and the clinical impact was even larger in patients with NNRTI PDR. Similar results were observed both in adults and in children.

In most study settings, PDR is largely driven by resistance to the NNRTI drug class, which likely explains the strong clinical impact of PDR, even when it is loosely defined as any resistance mutations detected at time of ART initiation. While the risk of death was not increased in people with PDR, the lack of effect may be due to the short duration of follow-up.

In a randomized study in Kenya, Chung and colleagues report less frequent VF in people prescribed EFV-based ART than among those given NVP-based ART (OR, 0.37; *P* < .0001) [7]. Although it is conceivable that the impact of PDR may be milder if EFV is part of the first-line ART, this systematic review suggests that the impact remained present in patients receiving EFV-based ART (OR, 3.77).

In addition, it has been suggested that the predictive value for virologic failure in people initiating EFV/XTC/TDF with NNRTI PDR with or without NRTI PDR may be different [44]. In a large South African study, the presence of NNRTI plus NRTI mutations vs no PDR was associated with longer time to VS (adjusted hazard ratio [aHR], 0.32 [95% CI, .12–.86]), whereas there was no difference in outcome between those with only NNRTI mutations vs no PDR (aHR, 1.05 [95% CI, .82–1.34]) at the 5% or 20% threshold [8].

This result seems at odds with our findings showing an increased risk of VF in people with NNRTI PDR receiving TDF/XTC/EFV. The observed difference may be explained by the use of different definitions for NNRTI PDR. Derache et al [8] assessed the impact of NNRTI PDR, defined as the presence of only NNRTI mutations (ie, excluding sequences with both NRTI and NNRTI mutations), whereas we assessed the impact of NNRTI PDR irrespective of whether NNRTI mutations were present with NRTI mutations.

In an attempt to improve comparability of study results, Derache et al repeated their analysis using our study definition of resistance, which was applied to all of the 6 studies contributing to our meta-analysis. Derache et al found that among individuals with NNRTI PDR (irrespective of whether NNRTI mutations were present with NRTI mutations), there was a stronger trend, albeit non–statistically significant, toward a reduced likelihood of achieving VS (HR, 0.87 [.66–1.14]) [8]. This result was included in our meta-analysis and is in line with the directionality of the effect seen in the other studies. Of note, the VL cutoff used by Derache was <50 copies/mL, which is less sensitive than the cutoff of >1000 copies/mL employed in the other 5 studies included in this analysis, and may also explain the discrepancy.

An increased likelihood of VF among people with PDR on EFV/XTC/TDF was also observed in 2 recent large studies: a the phase 3, randomized clinical trial comparing TAF/FTC/DTG, TDF/FTC/DTG or TDF/FTC/EFV for first-line treatment of HIV-1 infection (ADVANCE) [44], in which VS among those with and without PDR was 65% and 85%, respectively (*P* < .001), and a case-cohort substudy of the HIV/AIDS Drug Resistance Surveillance Study (ADReSS), enrolling 1000 patients initiating first-line efavirenz/emtricitabine/tenofovir in KwaZulu-Natal, South Africa [45], in which PDR was associated with a 3-fold greater risk of VF (*P* = .002).

Surprisingly, the ADVANCE study also showed a greater risk of VF among people with PDR vs no PDR receiving DTG-based ART [44]. These results merit additional investigation, as explanations for this observation remain to be elucidated.

Taken together, the evidence points to the overall negative impact of PDR on treatment outcomes in programmatic settings where pretreatment HIV-DR testing is unavailable, and highlights the clinical relevance of results obtained from recent national PDR surveys in LMICs. Of 18 countries reporting data to WHO between 2014 and 2018 [2], 12 and 3 countries showed levels of NNRTI PDR >10% and >20%, respectively, and showed a significantly higher PDR in people restarting ART vs ART-naive starters.

WHO’s recommendation to move away from EFV-based ART in settings with high PDR levels is supported by cost-effectiveness analyses, which have predicted a substantially larger increase in the benefits of transitioning from EFV- to DTG-based generic formulations in countries in sub-Saharan Africa with prevalence of NNRTI PDR >10% [46].

While transition to DTG is set to overcome most challenges posed by high levels of NNRTI PDR, populations without access to DTG remain at risk, as they lack viable options for first-line treatment.

Ongoing use of NNRTI-based ART in LMICs can occur for several reasons:

1. The uptake of these WHO recommendations to use DTG or protease inhibitor–based ART in young children is rapidly increasing; however, too many children are still receiving NNRTI-based regimens globally. It is particularly concerning that not all infants are started on non-NNRTI-based regimens, as more than half of the infants newly diagnosed with HIV and ART naive in sub-Saharan Africa carry drug-resistant HIV before initiating treatment, ranging from 34% in Eswatini to 69% in Malawi as of 2019 [2]. The very high levels of PDR in young children, coupled with overall poor levels of VL suppression in this population, support the need to accelerate access to child-friendly non-NNRTI-based formulations to prevent poor treatment outcomes.
2. Despite WHO recommendations to provide DTG to all, DTG transition in women of childbearing potential (WCBP) has lagged behind in some countries due to concerns of potential risk of neural tube defects (NTDs) in babies born to mothers receiving DTG. As of July 2019, among 30 countries that included DTG as preferred first-line, 21 reported information on the eligible populations: 7 had a policy of providing non-DTG-based ART to all WCBP, 4 required women to receive contraception (any type), 6 required use of long-term contraception, and in 4 countries DTG was provided to women based on informed choice after counseling on risk and benefits [47]. The slow uptake of DTG in WCBP is concerning as levels of NNRTI PDR in women exceed those observed in men. Among 11 LMICs contributing to WHO’s 2019 HIV-DR report with information disaggregated by sex, PDR to EFV/NVP among women initiating ART was ≥10% in 8 countries (4 in sub-Saharan Africa, 4 in Latin America) compared to 5 countries among men [48]. In pooled analysis of the 11 countries, PDR to EFV/NVP in women was nearly twice that in men (12.2% vs 6.3%; *P* < .0001) [48]. While use of DTG in WCBP in LMICs has been lagging, it is reassuring that updated evidence from the Tsepamo study showed that the prevalence of NTDs among infants born to women receiving DTG at conception seems to be stabilizing at approximately 0.2 percent, which is not statistically different from women taking non-DTG regimens. Consequently, DTG scale-up has now bounced back to match pre-NTD safety signal expectations of uptake and continues to scale up with large increases anticipated over the next few years [49].
3. DTG remains unavailable in a number of countries with a high burden of HIV infection. For example, access to DTG is currently limited in several upper-middle-income countries such as Russia, China, Colombia, and Trinidad and Tobago due to restrictions in the licensing agreement to allow manufacturers to produce generic DTG formulations [50]. Likewise, in these countries, pretreatment HIV-DR testing is not routinely available to inform regimen selection; thus, NNRTI-based regimens will continue to be used in first-line ART by a considerable proportion of people for some time. Cost-effectiveness modeling cost vs treatment outcomes, coupled with political will, is needed in these countries to accelerate DTG uptake or alternative non-NNRTI regimens.
4. To date, data on the safety of DTG are limited in subpopulations such as young children. Among the general population, data remain scarce regarding the toxicity of DTG within large-scale national treatment programs. Higher incidence of central nervous system adverse drug reactions such as insomnia, hyperglycemia, and weight gain have been reported among individuals receiving DTG.

Our findings suggest caution in using NNRTI-based ART in cases where DTG is not suitable or available.

Our results should be interpreted in light of the following limitations. First, data were drawn mostly from regression estimates, with composition of the regression models (factors controlled for) varying across studies; in some instances, unadjusted estimates were used. Second, concerns about selection bias, comparability of cases and controls, and attrition limit the certainty of the overall body of evidence. It is, however, unlikely that these sources of bias have led to profound under- or overestimation of the effect of PDR on treatment outcomes. Many of these sources of uncertainty are inherent to studies of PDR, as PDR is not amenable to randomization, given that there is an ethical imperative to act upon knowledge of PDR. In all of the included studies, HIV-DR testing was performed retrospectively on stored samples. Third, the exclusion of data from studies using next-generation sequencing technology with limits of detection <20% or from studies using point mutation assays may have led to the exclusion of potentially informative studies; however, this approach was adopted to reflect generalizable interpretation of resistance data. Fourth, given that studies did not report outcomes separately for individuals with NNRTI mutations only vs NRTI and NNRTI mutations, or whether such mutations, when detected, were on the same genome, we were unable to assess the impact of single vs dual class resistance or of mutation linkage. However, while it is unlikely that this had a large impact on our results because very few new ART initiators have both NRTI and NNRTI HIV-DR mutations, the possibility that single-class NNRTI PDR is not predictive of virological failure of treatment with EFV/XTC/TDF should be investigated in future studies. Fifth, as raw sequence data were unavailable to us for reanalysis, we are unable to comment on the possible impact of specific mutations, polymorphisms, or HIV subtype on study outcomes. Sixth, very few studies reported the outcome of death; thus, mortality outcome in this analysis should be regarded with caution.

Last, in the subanalysis restricted to studies with people receiving EFV/XTC/TDF, EFV was dosed at 600 mg daily in all studies except 1, in which the dose was 400 mg daily [22]. We are unable to assess whether the clinical impact of NNRTI PDR is worse in people using EFV 400 mg vs 600 mg daily.

The strength of this systematic review lies in the exhaustiveness of the search and the diverse geographic locations in which the studies were conducted, suggesting a wide generalizability. To maximize data comparability of the EFV and EFV/XTC/TDF subgroup analysis, we contacted study authors, who reanalyzed their data using common definition of resistance.

In conclusion, this systematic review and meta-analysis provides evidence of poorer treatment outcomes—that is, higher risk of VF, acquisition of new resistance mutation, or ART switch or discontinuation—in both adults and children with PDR (either PDR or NNRTI PDR) initiating NNRTI-containing regimens. This conclusion holds true in people receiving EFV/XTC/TDF.

Our findings are significant due to documented high levels of NNRTI PDR and the lack of PDR testing in many LMICs, particularly in situations where a substantially large proportion of the population has limited access to or poor tolerability of currently recommended DTG-based first-line ART.

These results have informed the WHO guidelines on the public health response to PDR and the 2019 WHO recommendations on first- and second-line ART, indicating to move away from NNRTI in settings with high levels of PDR.

## Supplementary Data

Supplementary materials are available at *The Journal of Infectious Diseases* online. Consisting of data provided by the authors to benefit the reader, the posted materials are not copyedited and are the sole responsibility of the authors, so questions or comments should be addressed to the corresponding author.

jiaa683_suppl_Supplementary_Appendix-1

jiaa683_suppl_Supplementary_Appendix-2

jiaa683_suppl_Supplementary_Appendix-3

## Notes

***Author contributions.*** S. B. designed the study, helped find articles, interpreted the data, and drafted the manuscript. T. A. led the search, extracted data, and created the tables. A. B., A. L., L. H., and A. W. screened studies, extracted data, and conducted analyses. L. M. extracted data, conducted the meta-analyses, and created the figures. S. B., L. H., N. P., S. I., and M. R. J. helped find articles, supported the screening, and extracted and interpreted data. A. W., S. B., S. I., and L. H. interpreted data and gave feedback on initial analyses. L. H., S. A., C. I., A. D., E. D., and S. I. contributed to the review with original analysis of their data. All authors reviewed and contributed to the final manuscript.

***Financial support.*** This work was supported by the WHO, through a grant from the Bill & Melinda Gates Foundation. L. H. was supported by The Netherlands Organisation for Health Research and Development (ZonMW/WOTRO; grant number 205300004).

***Potential conflicts of interest.*** C. I. has received honoraria for consulting services, conference support, and research grants from Gilead Sciences. All other authors report no potential conflicts of interest.
