# Supplementary material for: Clinical Impact of Pretreatment Human Immunodeficiency Virus Drug Resistance in People Initiating Nonnucleoside Reverse Transcriptase Inhibitor–Containing Antiretroviral Therapy: A Systematic Review and Meta-analysis
Source: J Infect Dis. 2020 Nov 17;224(3):377–88. doi: 10.1093/infdis/jiaa683 (PMC8328216; doi:10.1093/infdis/jiaa683)
Supplement: jiaa683_suppl_Supplementary_Appendix-1 [file jiaa683_suppl_supplementary_appendix-1.docx]

**Appendix 1:** **Search strategies, data extraction, and analysis**

**Search strategies, data extraction**

We searched electronic databases (MEDLINE, Embase, Cochrane Central Register of Controlled Trials), trial registries (WHO International Clinical Trials Registry Platform; ClinicalTrials.gov; ARV-trials.com) and conference proceedings (Conference on Retroviruses and Opportunistic Infections; International AIDS Society Conference on HIV Pathogenesis, Treatment and Prevention). We manually searched the reference lists of all pertinent reviews and included studies. We contacted experts in the field for unpublished and ongoing studies. We did not place any restrictions on language. We used antiretroviral terms from the Cochrane Infectious Diseases Group search strategies and additional terms for randomized trials, cohort studies and resistance testing, including Medical Subject Heading (MeSH) terms.

We used a web-based platform to screen and extract data (DistillerSR, Evidence Partners, Ottawa, Canada). Two of six investigators (TA, AB, AL, LH, AW, or LM) independently screened the titles and abstracts. Full articles of potentially relevant studies were scrutinised for eligibility and tagged for inclusion. These investigators resolved any disagreements regarding study inclusion by discussion until consensus was achieved. When consensus could not be reached, a third author arbitrated (SB, MJ, NP). We contacted the authors of included studies to seek clarifications on missing or unclear information. Two (TA, AB, LH, AL or LM) of six authors independently extracted data, including bibliometric information, clinical outcomes, duration of follow-up, sample size, PDR prevalence in the study population, previous exposure to antiretroviral drugs, ART regimen, and data pertaining to the risk or odds of experiencing any of the outcomes of interests in raw (in tables), crude (odds ratios) or adjusted form (adjusted odds ratios). We sought additional data from secondary publications of the same study or supplementary files.

Authors of studies including patients receiving NNRTIs were contacted and invited to extract and share data on the correlation between PDR and treatment outcomes in subpopulation receiving EFV-based ART and, where available, EFV/XTC/TDF, using standardized definition of resistance.

Two of six investigators independently screened the titles and abstracts. These investigators resolved any disagreements regarding study inclusion by discussion until consensus was achieved. When consensus could not be reached, a third author arbitrated. We contacted the authors of included studies to seek clarifications on missing or unclear information. We sought additional data from secondary publications of the same study or supplementary files.

**CENTRAL**

| ID | Search |
| --- | --- |
| #1 | MeSH descriptor: [HIV Infections] explode all trees |
| #2 | MeSH descriptor: [HIV] explode all trees |
| #3 | hiv or hiv-1* or hiv-2* or hiv1 or hiv2 or (hiv near infect*) or (human immunodeficiency virus) or (human immunedeficiency virus) or (human immune-deficiency virus) or (human immuno-deficiency virus) or (human immune deficiency virus) or (human immuno deficiency virus) or (acquired immunodeficiency syndrome) or (acquired immunedeficiency syndrome) or (acquired immuno-deficiency syndrome) or (acquired immune-deficiency syndrome) or (acquired immun* deficiency syndrome) |
| #4 | MeSH descriptor: [Lymphoma, AIDS-Related] this term only |
| #5 | MeSH descriptor: [Sexually Transmitted Diseases, Viral] this term only |
| #6 | #1 or #2 or #3 or #4 or #5 |
| #7 | [mh ^genotype] or genotype:ti,ab,kw or [mh "genotypic techniques"] or genotypic:ti,ab,kw or genotyping:ti,ab,kw or genotypical:ti,ab,kw (Word variations have been searched) |
| #8 | [mh ^phenotype] or phenotype:ti,ab,kw or phenotypic:ti,ab,kw or phenotyping:ti,ab,kw or phenotypical:ti,ab,kw (Word variations have been searched) |
| #9 | (resistance or resistant):ti,ab,kw (Word variations have been searched) |
| #10 | #7 or #8 or #9 |
| #11 | (test or tests or tested or testing or assay or assays):ti,ab,kw (Word variations have been searched) |
| #12 | #10 and #11 |
| #13 | [mh "drug resistance"] or resistance:ti,ab,kw or resistant:ti,ab,kw (Word variations have been searched) |
| #14 | #6 and #12 and #13 Publication Year from 1989 to 2017 |

**PubMed**

| Search | Query |
| --- | --- |
| #1 | Search (((#4 AND #10 AND #11 AND #12))) AND ("1989/01/01"[Date - Publication] : "2017/02/01"[Date - Publication]) |
| #2 | Search (#4 AND #10 AND #11 AND #12) |
| #3 | Search (randomized controlled trial [pt] OR controlled clinical trial [pt] OR randomized [tiab] OR placebo [tiab] OR drug therapy [sh] OR randomly [tiab] OR trial [tiab] OR groups [tiab] OR cohort studies[mh:noexp] OR cohort[tiab] OR longitudinal studies[mh:noexp] OR longitudinal[tiab] OR follow-up studies[mh:noexp] OR follow-up[tiab] OR followup[tiab] OR prospective studies[mh:noexp] OR prospective[tiab] OR retrospective studies[mh:noexp] OR retrospective[tiab] OR epidemiologic studies[mh:noexp]) NOT (animals [mh] NOT humans [mh]) |
| #4 | Search (drug resistance[mh] OR resistance[tiab] OR resistant[tiab]) |
| #5 | Search (#8 AND #9) |
| #6 | Search (test[tiab] OR tests[tiab] OR testing[tiab] OR tested[tiab] OR assay[tiab] OR assays[tiab]) |
| #7 | Search (#5 OR #6 OR #7) |
| #8 | Search (resistance[tiab] OR resistant[tiab]) |
| #9 | Search (phenotype[mh:noexp] OR phenotype[tiab] OR phenotypic[tiab] OR phenotyping[tiab] OR phenotypical[tiab]) |
| #10 | Search (genotype[mh:noexp] OR genotypic techniques[mh] genotype[tiab] OR genotypic[tiab] OR genotyping[tiab] OR genotypical[tiab]) |
| #11 | Search (HIV Infections[MeSH] OR HIV[MeSH] OR hiv[tiab] OR hiv-1*[tiab] OR hiv-2*[tiab] OR hiv1[tiab] OR hiv2[tiab] OR hiv infect*[tiab] OR human immunodeficiency virus[tiab] OR human immunedeficiency virus[tiab] OR human immuno-deficiency virus[tiab] OR human immune-deficiency virus[tiab] OR ((human immun*[tiab]) AND (deficiency virus[tiab])) OR acquired immunodeficiency syndrome[tiab] OR acquired immunedeficiency syndrome[tiab] OR acquired immuno-deficiency syndrome[tiab] OR acquired immune-deficiency syndrome[tiab] OR ((acquired immun*[tiab]) AND (deficiency syndrome[tiab])) |

**EMBASE**

| No. | Query |
| --- | --- |
| #17 | #1 AND #7 AND #8 AND #16 |
| #16 | #11 NOT #15 |
| #15 | #12 NOT #14 |
| #14 | #12 AND #13 |
| #13 | 'human'/de OR 'normal human'/de OR 'human cell'/de |
| #12 | 'animal'/de OR 'animal experiment'/de OR 'invertebrate'/de OR 'animal tissue'/de OR 'animal cell'/de OR 'nonhuman'/de |
| #11 | #9 OR #10 |
| #10 | 'randomized controlled trial'/de OR 'randomized controlled trial' OR random*:ab,ti OR trial:ti OR allocat*:ab,ti OR factorial*:ab,ti OR placebo*:ab,ti OR assign*:ab,ti OR volunteer*:ab,ti OR 'crossover procedure'/de OR 'crossover procedure' OR 'double-blind procedure'/de OR 'double-blind procedure' OR 'single-blind procedure'/de OR 'single-blind procedure' OR (doubl* NEAR/3 blind*):ab,ti OR (singl*:ab,ti AND blind*:ab,ti) OR crossover*:ab,ti OR cross+over*:ab,ti OR (cross NEXT/1 over*):ab,ti |
| #9 | 'prospective study'/de OR prospective:ab,ti OR 'cohort analysis'/de OR cohort:ab,ti OR 'longitudinal study' OR longitudinal:ab,ti OR 'experimental design'/de OR 'retrospective study'/de OR retrospective:ab,ti OR 'follow up'/de OR 'follow+up':ab,ti OR followup:ab,ti |
| #8 | 'drug resistance'/exp OR resistance:ab,ti OR resistant:ab,ti |
| #7 | #5 AND #6 |
| #6 | test:ab,ti OR tests:ab,ti OR testing:ab,ti OR tested:ab,ti OR assay:ab,ti OR assays:ab,ti |
| #5 | #2 OR #3 OR #4 |
| #4 | resistance:ab,ti OR resistant:ab,ti |
| #3 | 'phenotype'/de OR phenotype:ab,ti OR phenotypic:ab,ti OR phenotyping:ab,ti OR genotypical:ab,ti |
| #2 | 'genotyping technique'/de OR genotype:ab,ti OR genotypic:ab,ti OR genotyping:ab,ti OR genotypical:ab,ti |
| #1 | 'human immunodeficiency virus infection'/exp OR 'human immunodeficiency virus'/exp OR 'human immunodeficiency virus':ab,ti OR 'human immuno+deficiency virus':ab,ti OR 'human immunedeficiency virus':ab,ti OR 'human immune+deficiency virus':ab,ti OR hiv:ab,ti OR 'hiv-1':ab,ti OR 'hiv-2':ab,ti OR 'acquired immunodeficiency syndrome':ab,ti OR 'acquired immuno+deficiency syndrome':ab,ti OR 'acquired immunedeficiency syndrome':ab,ti OR 'acquired immune+deficiency syndrome':ab,ti |

**Data analysis**

*Assessment of risk of bias.* This systematic review is reported according to PRISMA.^17^ Two investigators assessed the methodological quality of all studies with the Newcastle Ottawa Scales.^18^

*Statistical analysis.* The primary outcome of the analysis was VF. Studies providing data on the composite outcome of *VF or death* were pooled with studies providing VF outcomes, as well as were analysed separately. Secondary outcomes were death, discontinuation of ART, switch to a non-NNRTI-based regimen, and incident drug resistance mutations.

Odds ratios (OR) or hazard ratios (HR) with corresponding 95% confidence intervals (CI) were extracted. Crude ORs were computed when not reported in the paper, but underlying data were available. In this analysis, given the inclusion of observational studies, adjusted data from full regression models (adjusting for all relevant variables) were preferred for analysis over unadjusted data. In addition, studies reporting data using time-to-event outcomes (e.g. time to failure) were converted to binary outcomes (e.g. VF). HR and OR were converted from positive to negative for pooling to reflect the relative effect of having PDR versus not having PDR.

All included studies were examined to determine if there was any overlap in cohorts. Only unique outcomes from overlapping cohorts were used. Two separate publications, one reporting on VF and the other on emergence of new drug resistance mutations, would both be included even if they analysed data from the same patients.

We pooled similar measures of effect where appropriate. Only data from sufficiently similar studies were combined (similar participants, interventions, comparisons and outcomes) in a random effects meta-analysis. We used the generic inverse variance approach to incorporate adjusted effect estimates.

Statistical heterogeneity was assessed using the Chi-squared test for homogeneity with a level of significance, alpha = 0.10 and the I^2^ statistic to quantify inconsistency. Publication bias was assessed using a funnel plot for outcomes with ten or more studies.

We conducted subgroup analyses by regimens (NNRTI-based, EFV-based, regimen containing EFV/XTC/TDF) and populations (children versus adults).

We analysed the data using Stata/IC 16.0 for Windows,^19^ and WINPEPI,^20^ and present the results as effect measures and 95% CIs.
