# Supplementary material for: Clinical Impact of Pretreatment Human Immunodeficiency Virus Drug Resistance in People Initiating Nonnucleoside Reverse Transcriptase Inhibitor–Containing Antiretroviral Therapy: A Systematic Review and Meta-analysis
Source: J Infect Dis. 2020 Nov 17;224(3):377–88. doi: 10.1093/infdis/jiaa683 (PMC8328216; doi:10.1093/infdis/jiaa683)
Supplement: jiaa683_suppl_Supplementary_Appendix-2 [file jiaa683_suppl_supplementary_appendix-2.docx]

**Appendix 2: Characteristics of excluded studies**

| **Reason for exclusion** | **Study reference** |
| --- | --- |
| **No baseline resistance test** | 1. Pinoges, L, Schramm, B, Poulet, E, Balkan, S, Szumilin, E *et al*: **Risk factors and mortality associated with resistance to first-line antiretroviral therapy: Multicentric cross-sectional and longitudinal analyses**. *Journal of Acquired Immune Deficiency Syndromes* 2015, **68**(5):527-535. 2. Fethi, T, Asma, J, Amine, S M, Ammar A, E. G B, Taoufik, B C *et al*: **Effects on immunological and virological outcome of patients using one protease inhibitor or one non-nucleoside reverse transcriptase inhibitor in a triple antiretroviral therapy: Normal clinical practice versus clinical trial findings**. *Current HIV Research* 2005, **3**(3):271-276. 3. Luebbert J, Tweya H, Phiri S, Chaweza T, Mwafilaso J, Hosseinipour MC, Ramroth H, Schnitzler P, Neuhann F: **Virological failure and drug resistance in patients on antiretroviral therapy after treatment interruption in Lilongwe, Malawi**. Clin Infect Dis 2012, 55(3):441-448. 4. Mollan KR, Daar ES, Tierney C, Dalai S, Balamane M, Sax PE, Collier AC, Fischl MA, Lalama CM, Bosch RJ et al: **Reverse transcriptase (RT) and protease (PR) drug resistance mutations (DRM) and evolution at virological failure (VF) in AIDS clinical trials group (ACTG) study A5202**. Antiviral Therapy 2011, 16:A124. 5. Wadonda-Kabondo N, Bennett D, van Oosterhout JJ, Moyo K, Hosseinipour M, Devos J, Zhou Z, Aberle-Grasse J, Warne TR, Mtika C *et al*: **Prevalence of HIV drug resistance before and 1 year after treatment initiation in 4 sites in the Malawi antiretroviral treatment program**. *Clin Infect Dis* 2012, **54 Suppl 4**:S362-368. |
| **No comparison of interest** | 1. Duarte, H, Almeida, P, Alcobia, A: **One year evaluation of treatment in a cohort of treatment-naive patients: Compliance with national and international guidelines?** *European Journal of Hospital Pharmacy* 2014, **21**:A148-A149. 2. Marcelin, A G, Soulié, C, Assoumou, L, Ghosn, J, Duvivier, C *et al*: **NRTI-sparing regimen (NNRTI+PI) was more likely to be associated with drug resistance compared with NNRTI+NRTI or PI+NRTI in the randomized ANRS 121 trial**. *Antiviral Therapy* 2009, **14**(5):A56. 3. Ndembi, N, Goodall, R L, Dunn, D T, McCormick, A, Burke, A *et al*: **Viral rebound and emergence of drug resistance in the absence of viral load testing: A randomized comparison between zidovudine-lamivudine plus nevirapine and zidovudine-lamivudine plus abacavir**. *Journal of Infectious Diseases* 2010, **201**(1):106-113. 4. Rhee, S Y, Fessel, W J, Liu, T F, Hurley, L, Klein, D et al: **Virologic outcome associated with genotypeguided therapy in patients with transmitted HIV-1 drug resistance (TDR) between 2003 and 2006.** Antiviral Therapy 2009, 14(5):A62. 5. Fogel JM, Hudelson SE, Ou SS, Hart S, Wallis C, Morgado MG, Saravanan S, Tripathy S, Hovind L, Piwowar-Manning E *et al*: **Brief Report: HIV Drug Resistance in Adults Failing Early Antiretroviral Treatment: Results From the HIV Prevention Trials Network 052 Trial**. *J Acquir Immune Defic Syndr* 2016, **72**(3):304-309. 6. Ferrer E, Podzamczer D, Arnedo M, Fumero E, McKenna P, Rinehart A, Pérez JL, Barberá MJ, Pumarola T, Gatell JM: **Genotype and Phenotype at Baseline and at Failure in Human Immunodeficiency Virus–Infected Antiretroviral-Naive Patients in a Randomized Trial Comparing Zidovudine and Lamivudine plus Nelfinavir or Nevirapine**. *The Journal of Infectious Diseases* 2003, **187**(4):687-690. 7. Lockman S, Hughes M, Sawe F, Zheng Y, McIntyre J, Chipato T, Asmelash A, Rassool M, Kimaiyo S, Shaffer D *et al*: **Nevirapine- Versus Lopinavir/Ritonavir-Based Initial Therapy for HIV-1 Infection among Women in Africa: A Randomized Trial**. *PLOS Medicine* 2012, **9**(6):e1001236. 8. Grant RM, Hecht FM, Warmerdam M, Liu L, Liegler T, Petropoulos CJ, Hellmann NS, Chesney M, Busch MP, Kahn JO: **Time trends in primary HIV-1 drug resistance among recently infected persons**. *JAMA* 2002, **288**(2):181-188. 9. Poggensee G, Kucherer C, Werning J, Somogyi S, Bieniek B, Dupke S, Jessen H, Hamouda O: **Impact of transmission of drug-resistant HIV on the course of infection and the treatment success. Data from the German HIV-1 Seroconverter Study**. *HIV Med* 2007, **8**(8):511-519. |
| **No resistance data** | 1. Leonard, H, Gonzalez, A, Burch, L, Marshall, N, Levitt, D *et al*: **Mother-to-child transmission (MTCT) of HIV-almost a thing of the past? A cohort study of HIV-positive women starting antiretroviral drugs in pregnancy**. *HIV Medicine* 2014, **15**:58. |
| **Use of genotyping techniques not comparable to Sanger** | 1. Seclén, E, Gonzalez, M, Martín C, L, Gellermann, H, Cairns, V *et al*: **Impact of baseline HIV-1 tropism on viral response and CD4 gains in antiretroviral-naïve patients**. *Journal of the International AIDS Society* 2010, **13**. 2. Peuchant O, Thiebaut R, Capdepont S, Lavignolle-Aurillac V, Neau D, Morlat P, Dabis F, Fleury H, Masquelier B: **Transmission of HIV-1 minority-resistant variants and response to first-line antiretroviral therapy**. *AIDS* 2008, **22**(12):1417-1423. 3. Jourdain, G, Wagner, T A, Ngo Giang H, N, Sirirungsi, W, Klinbuayaem, V *et al*: **Association between detection of HIV-1 DNA resistance mutations by a sensitive assay at initiation of antiretroviral therapy and virologic failure**. *Clinical Infectious Diseases* 2010, **50**(10):1397-1404. 4. Katzenstein, D A, Bosch, R J, Hellmann, N, Wang, N, Bacheler, L et al: **Phenotypic susceptibility and virological outcome in nucleoside-experienced patients receiving three or four antiretroviral drugs.** AIDS (London, England) 2003, 17(6):821-830. 5. Chung, M H, Beck, I A, Dross, S, Tapia, K, Kiarie, J N et al: Oligonucleotide ligation assay detects HIV drug resistance associated with virologic failure among antiretroviral-naïve adults in Kenya. Journal of Acquired Immune Deficiency Syndromes 2014. 6. Kanthula R, Beck I, Dyk GV, Silverman R, Olson S, Salyer C, Cassol S, Rossouw T, Frenkel L: **Persistence of HIV Drug Resistance Among South African Children Exposed to Nevirapine Prophylaxis for Prevention of Mother-to-Child-Transmission (pMTCT)**. *Open Forum Infectious Diseases* 2015, **2**(suppl_1):466-466. 7. Soria J., Mugruza R., Levine M., León S.R., Arévalo J., Ticona E., Beck I.A., Frenkel L.M. **Pretreatment HIV Drug Resistance and Virologic Outcomes to First-Line Antiretroviral Therapy in Peru**. *AIDS Research and Human Retroviruses* 2019 **35**(2):150-154. 8. Hassan A.S., Bibby D.F., Mwaringa S.M., Agutu C.A., Ndirangu K.K., Sanders E.J., Cane P.A., Mbisa J.L., Berkley J.A. **Presence, persistence and effects of pretreatment HIV-1 drug resistance variants detected using next generation sequencing: A Retrospective longitudinal study from rural coastal Kenya**. *PLoS ONE* (2019) **14**(2): e0210559. |
| **Pretreatment mutations not reported in the target group of patients; or not adequately reported** | 1. Shet A, Berry L, Mohri H, Mehandru S, Chung C, Kim A, Jean-Pierre P, Hogan C, Simon V, Boden D *et al*: **Tracking the prevalence of transmitted antiretroviral drug-resistant HIV-1: A decade of experience**. *Journal of Acquired Immune Deficiency Syndromes* 2006, **41**(4):439-446. 2. Pillay D, Porter K: **The impact of transmitted drug resistance on the natural history of HIV infection and response to first-line therapy.** AIDS 2006, 20(1):21-28. 3. Jakobsen MR, Tolstrup M, Sogaard OS, Jorgensen LB, Gorry PR, Laursen A, Ostergaard L: **Transmission of HIV-1 drug-resistant variants: prevalence and effect on treatment outcome**. Clin Infect Dis 2010, 50(4):566-573. 4. Mackie NE, Dunn DT, Dolling D, Garvey L, Harrison L, Fearnhill E, Tilston P, Sabin C, Geretti AM; UK HIV Drug Resistance Database; UK CHIC study. **The impact of HIV-1 reverse transcriptase polymorphisms on responses to first-line nonnucleoside reverse transcriptase inhibitor-based therapy in HIV-1-infected adults**. *AIDS* 2013, **27**(14):2245-53. |
| **On acquired drug resistance** | 1. Hingankar NK, Thorat SR, Deshpande A, Rajasekaran S, Chandrasekar C, Kumar S, Srikantiah P, Chaturbhuj DN, Datkar SR, Deshmukh PS *et al*: **Initial virologic response and HIV drug resistance among HIV-infected individuals initiating first-line antiretroviral therapy at 2 clinics in Chennai and Mumbai, India**. *Clin Infect Dis* 2012, **54 Suppl 4**:S348-354. |
| **Patients not on NNRTI** | 1. Harzic M, Pellegrin I, Deveau C, Chaix ML, Dubeaux B, Garrigue I, Ngo N, Rouzioux C, Goujard C, Hoen B *et al*: **Genotypic drug resistance during HIV-1 primary infection in France (1996-1999): frequency and response to treatment**. *AIDS* 2002, **16**(5):793-796. 2. Kulkarni, R, Abram, M E, Rhee, M S, Fordyce, M W, Szwarcberg, J et al: **Week 144 resistance analyses of the phase 3 EVG/COBI/FTC/TDF studies.** Topics in Antiviral Medicine 2014, 22:288. 3. Keita A., Sereme Y., Pillet S., Coulibaly S., Diallo F., Pozzetto B., Thiero T.A., Bourlet T. **Impact of HIV-1 primary drug resistance on the efficacy of a first-line antiretroviral regimen in the blood of newly diagnosed individuals in Bamako, Mali.** *Journal of Antimicrobial Chemotherapy* 2019, **74**(1):165-171. |
| **Patients were on ART** | 1. Vray, M, Meynard, J L, Dalban, C, Morand J, L, Clavel, F *et al*: **Predictors of the virological response to a change in the antiretroviral treatment regimen in HIV-1-infected patients enrolled in a randomized trial comparing genotyping, phenotyping and standard of care (Narval trial, ANRS 088)**. *Antiviral therapy* 2003, **8**(5):427-434. 2. Armenia D, Di Carlo D, Calcagno A, Vendemiati G, Forbici F, Bertoli A, Berno G, Carta S, Continenza F, Fedele V et al: **Pre-existent NRTI and NNRTI resistance impacts on maintenance of virological suppression in HIV-1-infected patients who switch to a tenofovir/emtricitabine/rilpivirine single-tablet regimen.** Journal of antimicrobial chemotherapy 2017, 72(3):855-865. |
