# Supplementary material for: Clinical Impact of Pretreatment Human Immunodeficiency Virus Drug Resistance in People Initiating Nonnucleoside Reverse Transcriptase Inhibitor–Containing Antiretroviral Therapy: A Systematic Review and Meta-analysis
Source: J Infect Dis. 2020 Nov 17;224(3):377–88. doi: 10.1093/infdis/jiaa683 (PMC8328216; doi:10.1093/infdis/jiaa683)
Supplement: jiaa683_suppl_Supplementary_Appendix-3 [file jiaa683_suppl_supplementary_appendix-3.docx]

**Appendix 3: Risk of bias in included studies using the Newcastle Ottawa Scale**

Appendix 3a: Cohort studies

|  | **Selection** | | | | **Comparability** | | **Exposure/Outcome** | | | **Total** |
| --- | --- | --- | --- | --- | --- | --- | --- | --- | --- | --- |
| **Study** | **Representativeness of the exposed cohort** | **Selection of the non exposed cohort** | **Ascertainment of exposure** | **Demonstration that outcome of interest was not present at start of study** | **Important factor** | **Additional factor** | **Assessment of outcome** | **Sufficiently long follow-up** | **Adequacy of follow-up** |  |
| **Avila-Rios 2016** | * | * | * | * | * | * | * | * | * | 9 |
| **Bannister 2008** |  | * | * | * | * | * | * | * | * | 8 |
| **Bansi 2010** | * | * | * | * | * | * | * | * |  | 8 |
| **Boender 2015** | * | * | * | * | * | * | * | * |  | 8 |
| **Boerma 2016** | * | * | * | * | * | * | * | * | * | 9 |
| **Borroto-Esoda 2007** |  | * | * | * | * | * | * | * | * | 8 |
| **Chaix 2007** | * | * | * | * | * | * | * | * | * | 9 |
| **Clutter 2016** | * | * | * | * | * | * | * | * | * | 9 |
| **Coelho 2018** | * | * | * | * | * | * | * | * | * | 9 |
| **Crowell 2015** | * | * | * | * | * | * | * | * | * | 9 |
| **Derache 2019** |  | * | * | * | * | * | * | * |  | 7 |
| **Hamers 2012** | * | * | * | * | * | * | * | * | * | 9 |
| **Hermans 2019** | * | * | * | * | * | * | * | * | * | 9 |
| **Hong 2015** | * | * | * | * | * | * | * | * |  | 8 |
| **Inzaule 2019** | * | * | * | * | * | * | * | * | * | 9 |
| **Kityo 2017** | * | * | * | * | * | * | * | * |  | 8 |
| **Lee 2014** | * | * | * | * |  |  | * | * |  | 6 |
| **Li 2015** | * | * | * | * | * | * | * | * | * | 9 |
| **Lockman 2010** | * | * | * | * | * |  | * | * | * | 8 |
| **McCluskey 2018** | * | * | * | * | * | * | * | * | * | 9 |
| **NAMSAL 2019** | * | * | * | * | * | * | * | * | * | 9 |
| **Ngo-Giang-Huong 2016** | * | * | * | * | * | * | * | * | * | 9 |
| **Palumbo 2010** | * | * | * | * | * | * | * | * | * | 9 |
| **Phanuphak 2014** | * | * | * | * | * | * | * | * |  | 8 |
| **Shet 2015** | * | * | * | * | * | * | * | * | * | 9 |
| **Taniguchi 2012** | * | * | * | * | * | * | * | * | * | 9 |
| **Thao 2018** | * | * | * | * | * | * | * | * | * | 9 |
| **Wittkop 2011** | * | * | * | * | * | * | * | * | * | 9 |
| **Zu Knyphausen 2014** | * | * | * | * | * | * | * | * |  | 8 |

Appendix 3b. Case control studies

|  | **Representativeness of the exposed cohort** | **Selection of the non exposed cohort** | **Ascertainment of exposure** | **Demonstration that outcome of interest was not present at start of study** | **Important factor** | **Additional factor** | **Assessment of outcome** | **Sufficiently long follow-up** | **Adequacy of follow-up** | **Total** |
| --- | --- | --- | --- | --- | --- | --- | --- | --- | --- | --- |
| **Kantor 2015** | * |  | * | * | ** | * | * | * | * | 8 |
| **Kuritzkes 2008** | * | * | * | * | * |  | * | * |  | 7 |
| **Lai 2012** | * | * |  |  | ** | * | * | * | * | 7 |
